# Characterization and prognosis of very elderly patients with anemia, cardio-cerebrovascular disease, and iron deficiency in four Portuguese hospitals

**DOI:** 10.1097/j.pbj.0000000000000212

**Published:** 2023-04-10

**Authors:** Rita Rego, Margarida Madeira, Eugeniu Gisca, Daniela Brigas, Ana Vigário, Arsénio Barbosa, Sílvia Pereira, Marta Soares, Ana Macedo

**Affiliations:** a Centro Hospitalar e Universitário do Porto (Internal Medicine Department), Porto, Portugal; b Centro Hospitalar de Setúbal (Internal Medicine Department), Setúbal, Portugal; c Hospital Garcia de Orta (Internal Medicine Department), Almada, Portugal; d Centro Hospitalar e Universitário de São João (Internal Medicine Department), Porto, Portugal; e Departamento de Ciências Biomédicas e Medicina, Universidade do Algarve, Faro, Portugal.

To the Editor

Cardiovascular disease (CVD) is the leading cause of death in Portugal.^1^ Anemia and iron deficiency (ID) are commonly present in the elderly and associated with adverse clinical outcomes.^2,3^ Portuguese data describing elderly and very elderly (VEP) patients are scarce, making it critical to characterize this population and obtain data on the actual dimension of their major clinical issues.

This study aimed to characterize and evaluate the prognosis of VEP with anemia and CVD in four Portuguese hospitals. Data from all patients admitted to the internal medicine wards in 2017 who fulfilled the study inclusion criteria, including being VEP (≥aged 80 years or older) and diagnosed with anemia and CVD at the time of admission, were obtained. Authorizations were obtained from all the ethics committees/institutional review boards from all the hospitals involved in this study.

Of the 16 446 patients admitted to the hospitals, 514 met the inclusion criteria. The median age of VEP was 86 years (interquartile range [IQR]: 83–89), 64.4% were female, and 81.5% resided in their homes. At admission, median hemoglobin was 10.7 g/dL [IQR: 9.57–11.5], median serum iron was 34 µg/dL [IQR: 25–50.5], median ferritin was 117.8 µg/dL [IQR: 59.73–246.5], and median transferrin saturation was 12% [IQR: 7–17.7]. The median hospitalization length was 8.5 days [IQR: 5–13]. The mortality rate for 18 months after discharge was 39.9%, and the 30-day readmission rate was 16.5%. Only 302 patients (59.0%) had serum iron and ferritin levels data. Globally, 7.0% of patients (n = 36) had ferritin levels below 30 µg/L, and 21% were on iron supplements. Mortality had a significant association with length of hospitalization (odds ratio [OR], 1.057, 95% confidence interval [CI]: 1.030–1.084, *P* = .001, calculated with multiple logistic regression model), age at admission (OR, 1.508, 95% CI: 1.033–2.201, *P* = .033, calculated with multiple logistic regression model), and hemoglobin levels (OR, 0.667, 95% CI: 0.414–1.073, *P* = .019, calculated with multiple logistic regression model). Regarding prognosis, the mortality rates of patients with and without ID were 25.7% and 46.5% (*P* = .028, calculated with the Fisher exact test).

Although anemia is a widely recognized risk factor for morbidity and mortality, it remains underestimated and unexplored in VEP. Accordingly, iron parameters were unavailable in this study for many patients hospitalized with anemia and CVD. Our study revealed that VEP at a higher age, with more prolonged hospitalizations and lower hemoglobin levels, were more likely to die within 18 months after discharge. ID was associated with a worse prognosis, but further studies are needed. Notably, the authors acknowledge the limitations of this study because of its retrospective nature and the consequent inability to control the recording of data in the clinical reports.

In conclusion, our study suggests that VEP with anemia and CVD may have higher mortality, which may be predicted from admission. We consider these results to warrant further investigation.

## Contributors

RR was responsible for data curation, draft preparation, and final revision. MM, AB, and SP were responsible for data curation and draft preparation. EG was responsible for conceptualization and draft preparation. DB and AV were responsible for methodology and draft preparation. MS was responsible for the investigation and draft preparation. AM was responsible for the formal analysis.

## Funding

Financial support and sponsorship: Vifor Pharma provided support for third-party medical writing assistance for this manuscript. The sponsor had no role in the study design, data analysis and interpretation, report writing, or the decision to submit this study.

## Conflicts of interest

The authors report no conflicts of interest.
